# Do weaner pigs need in-feed antibiotics to ensure good health and welfare?

**DOI:** 10.1371/journal.pone.0185622

**Published:** 2017-10-05

**Authors:** Alessia Diana, Edgar G. Manzanilla, Julia A. Calderón Díaz, Finola C. Leonard, Laura A. Boyle

**Affiliations:** 1 Pig Development Department, Teagasc Animal and Grassland Research and Innovation Centre, Moorepark, Fermoy, Co. Cork, Ireland; 2 School of Veterinary Medicine, UCD, Belfield, Dublin 4, Ireland; 3 Department of Animal Behaviour and Welfare, Institute of Genetics and Animal Breeding, Polish Academy of Sciences, ul. Postepu 36A, Jastrzębiec, Magdalenka, Poland; Michigan State University College of Veterinary Medicine, UNITED STATES

## Abstract

Antibiotics (AB) are used in intensive pig production systems to control infectious diseases and they are suspected to be a major source of antibiotic resistance. Following the ban on AB use as growth promoters in the EU, their prophylactic use in-feed is now under review. The aim of this study was to evaluate the effect of removing prophylactic in-feed AB on pig health and welfare indicators. Every Monday for six weeks, a subset of 70 pigs were weaned, tagged and sorted into two groups of 35 pigs according to weight (9.2 ± 0.6 kg). AB were removed from the diet of one group (NO, n = 6) and maintained in the other group (AB, n = 6) for nine weeks. Ten focal pigs were chosen per group. After c. five weeks each group was split into two pens of c.17 pigs for the following 4 weeks. Data were recorded weekly. Skin, tail, ear, flank and limb lesions of focal pigs were scored according to severity. The number of animals per group affected by health deviations was also recorded. The number of fights and harmful behaviours (ear, tail bites) per group was counted during 3×5min observations once per week. Data were analysed using mixed model equations and binomial logistic regression. At group level, AB pigs were more likely to have tail (OR = 1.70; *P* = 0.05) but less likely to have ear lesions than NO pigs (OR = 0.46; *P*<0.05). The number of ear bites (21.4±2.15 vs. 17.3±1.61; *P*<0.05) and fights (6.91±0.91 vs. 5.58±0.72; *P* = 0.09) was higher in AB than in NO pigs. There was no effect of treatment on health deviations and the frequency of these was low. Removing AB from the feed of weaner pigs had minimal effects on health and welfare indicators.

## Introduction

Antibiotics (AB) are an effective tool to control and treat infectious diseases in intensive production systems where high densities of animals facilitate the spread of pathogens [1–3]. However, there is an overreliance on their usage and farmers often see them as the only solution to disease challenges [4, 5, 6]. Prophylactic use of AB in feed around weaning is common on Irish pig farms [7, 8]. This could contribute to create AB resistance [9] which is a major global concern for human as well as animal health [10]. Over/misuse of AB is a particular problem when the medication is supplied in pig feed, whether for prophylactic or metaphylactic purposes, because it is not targeted towards sick individuals [11]. In the EU, it is likely that prophylactic use of AB will be banned in the near future [12]. Previous studies reported that replacing in-feed medication with alternative nutrition or management strategies has no detrimental effect on pig performance [13–16]. However, the consequences for animal health and welfare in current high density production systems are not well researched. Removal of prophylactic AB from pig feed could pose additional disease challenges thereby increasing the need for labour-intensive parenteral treatments and possibly resulting in poor compliance with dosing schedules. There could be other unintended or unforeseen challenges which may stress pigs and cause an increase in negative behaviours [17, 18] such as aggression [19] and tail, ear and flank biting [20, 21] all of which result in lesions and are major welfare problems in intensive production systems.

Therefore, the objective of this study was to determine the effect of removing prophylactic AB from pig feed and replacing it with parenteral treatments as needed, on pig health and welfare indicators, with particular focus on the performance and consequences of negative behaviours.

## Material and methods

### Animals and housing

All procedures were approved by the Teagasc Animal Ethics Committee (approval no. TAEC 40/2013). The study was carried out on a 300 sow farrow-to-finish commercial farm with a history of high antibiotic use. The farmer was willing to cooperate with the intensive data collection required during the period of study which took place between September and December, 2014 in Ireland. Some changes in management were introduced in the weeks prior to the start of the study including a reduction in stocking density, and an improved programme of environmental enrichment. These changes were made to ensure the farm was in compliance with Council Directive 2008/120/EC. Pigs were weaned at 28 ± 2 days of age and then transferred to first (28 to 60 d of age) and second (60 to 91 d of age) weaner stage accommodation, and finally to the finishing stage. The farm was positive for PRRSv, APP, *Mycoplasma hyopneumoniae* and influenza and had a clinical history of diseases in the past year including regular meningitis and diarrhoea episodes in the first weaner stage and respiratory outbreaks both in second stage weaners and finishers. Clinical problems were addressed with in-feed prophylactic medication with sulfadiazine-trimethoprim (TMS) and therapeutic levels of ZnO. For the trial, six batches of 140 Large White × Landrace crossbred pigs (840 pigs in total), which were tail docked after birth, were weaned over a six-week period. At weaning pigs were identified individually with an ear-tag and weighed. Thereafter they were assigned to four mixed sex pens of c. 35 pigs each according to weight (9.2 ± 0.6 kg). Four rooms were used in each of the first and second weaner stage accommodation, with two of the rooms in each stage being used twice and pen position within each room being alternated each week to minimise variation between treatments. Pens in each first weaner stage room (3 m L × 2.35 m W and 3 m L × 1.92 m W) had fully slatted plastic floors with solid plastic panel pen divisions. Pigs remained in these pens for almost 5 weeks. Pigs were transferred to the second weaner stage accommodation which comprised of eight pens per room. Each group of pigs was divided into two mixed sex pens of 15 to 17 pigs each with pen position being alternated each week to minimise variation between treatments. Pens (6 m L × 2.30 m W) had fully slatted plastic floors with solid plastic panel pen divisions and pigs remained in these pens for a further c. 4 weeks.

In both the first and second weaner stages, pens were furnished with two types of environmental enrichment: one rubber helicopter toy (EasyFix™ Rubber Products, Ballinasloe, Co. Galway, Ireland) and one strip (approximately 1 m long) of plastic based sack ‘cloth’ suspended from the side of the pen by a plastic cable tie. All pens had one nipple drinker and a soya bean meal, lactofeed (Volac, Ireland), wheat, barley based diet was provided (CP = 19%, DE = 14.4 MJ/kg). Pigs had *ad libitum* access to feed provided by a SPOTMIX liquid feeding system (Schauer Agrotronic GmbH, Prambachkirchen, Austria) from a trough with eight feeder spaces for the first weaner stage pens and four feeder spaces for the second weaner stage pens. The environmental temperature was automatically regulated and kept at the recommended average of 26°C for the first and 22.5°C for the second stage [22]. The room was artificially illuminated from 0800 till 1700 h.

### Experimental design and treatment

During both the first and second weaner stages (for a total of nine weeks), pigs received a diet where the in-feed antibiotic (sulfadiazine-trimethoprim, 14.4mg/kg BW/d; for 5 days/week) was randomly removed (NO; n = 12 pens) or maintained in the diet (AB; n = 12 pens). This medication pattern was prescribed by the practicing veterinarian 6 months before the study started. In both stages, pigs in both treatments were also administered parenteral amoxicillin (15 mg/kg BW) for 3 days when clinical signs of lameness, meningitis, severe respiratory disease or diarrhoea were detected by the farm staff.

Performance measurements were recorded for the entire cohort of 840 pigs. A subset of 70 pigs per each weaning week (two pens per week, one pen per treatment; NO—n = 6 pens and AB—n = 6 pens) was selected for the health and welfare assessment. In addition, twenty focal pigs were chosen every week from the subset of pigs (ten pigs per treatment; 120 focal pigs in total), on the basis of dispersion around the median weight of the pen and these animals received a second coloured ear-tag in the opposite ear for easy identification. Five focal pigs were maintained in each group when the groups were divided in two on transfer to the second stage weaner accommodation. The selection of a subset of pigs for the health and welfare assessment was necessary due to the labour-intensive nature of data collection (at focal and group level), which would have been more difficult to carry out on a large number of pigs.

### Group measurements

#### Production data and parenteral administration of antibiotics

Pigs were weighed as a group at the beginning of both the first (i.e. at weaning) and second weaner stages and the average pig weight was calculated. Daily feed intake of all groups within the entire cohort of pigs was automatically recorded by the SPOTMIX liquid feeding system. Feed dispensed was recorded at each valve, and each valve dispensed feed to two pens. Number of mortalities was recorded daily while the number of doses of parenteral injections of AB administered to pigs in each treatment was recorded by the farmer during the entire weaner stage (nine weeks). Stocking density was calculated on a weekly basis as number of pigs per m^2^. Average daily gain (ADG), average daily feed intake (ADFI) and feed conversion ratio (FCR) were also calculated for both the first and second weaner stages. These data were calculated based on the entire cohort of pigs (840 pigs in total) in order to provide an accurate figure of pig performance. Welfare, health and behavioural measurements of the subset of pigs were recorded once per week.

#### Welfare and health

Each group was observed for a 10 minute observation period once weekly on the same day. The observer stood outside the pen and recorded the number of animals affected by the health deviations defined in the Welfare Quality® protocol [23]: thermal comfort (shivering and panting), diseases [pumping 9heavy/laboured breathing), coughing, sneezing, rectal prolapse, rupture/hernia, diarrhoea and including neurological disorders and swollen limbs which are not included in the Welfare Quality® protocol] and tail, ear and flank lesions as well as lameness. The proportion of pigs per pen affected by these conditions was calculated.

#### Behaviour

The frequency of tail, ear, flank biting and aggressive (fights and head knock) behaviours was recorded on a weekly basis. During the first weaner stage, each group was observed during three, five minute long observation periods. When pigs were moved to the second weaner stage, each group was subdivided into two groups and behaviour observations were conducted in a similar manner except that groups were observed during three, 2.5 min long observation periods on the same day. Observations were made between 0900 and 1400 h on the same day each week as this period was pre-established as one of high activity in the pens.

### Focal pig measurements

#### Welfare and health

Welfare and health measurements were recorded once per week. At weaning, focal pigs were scored in the farrowing house before being mixed into the first weaner stage accommodation. Pigs were scored again 1 to 2 days after mixing and thereafter on a weekly basis until the end of the second weaner stage. This was achieved at the weaning inspection and until the end of the first weaner stage by catching the focal pigs and restraining them in their home pen; during the second weaner stage, the pigs’ heavier weight made catching and restraining them impossible so the scoring was carried out by following the focal pigs with a torch inside their pen. Additionally, focal pigs were weighed individually at weaning and at transfer to the second weaner stage.

#### Skin lesion scores

Skin lesions arising from aggression (i.e. scratches) were recorded on both sides of the body for all focal pigs by one trained observer. Lesions were scored on the back, hind quarter, flank, shoulder, neck and ear according to severity as per O’Driscoll *et al*. [24] on a 7-point scale where 0 represented no lesions; 1 = one minor lesion (scratches not penetrating the full dermal thickness i.e. superficial); 2 = more than one minor (superficial) or 1 red (i.e. deeper) lesion; 3 = more than one red lesion/deeper cuts; 4 = one deep red lesion/one dark scab; 5 = more than one deep red/dark scabs or one extensive lesion and 6 = more than one extensive lesion (severe laceration with infected wounds and/or dark scabs). Scores from all areas were summed to provide a total body lesion (TBL) score for each pig for each inspection date. Additionally, a front body lesion (FBL) score was also calculated by summing the scores of lesions on the ears, neck and shoulders whereas a rear body lesion score (RBL) was calculated by summing the scores of lesions on the flanks and hindquarters.

#### Tail lesions

Tail lesions (TL) were scored from 0 to 5 as per Harley *et al*. [25] on a 6-point scale where 0 = no evidence of tail biting; 1 = healed or mild lesions; 2 = evidence of chewing or puncture wounds, but no evidence of swelling; 3 = evidence of chewing or puncture wounds with swelling and signs of possible infection; 4 = partial loss of the tail and 5 = total loss of the tail.

#### Ear lesions

Ear lesions (EL) were scored using a scale from 0 to 3 where 0 = no lesion; 1 = mild lesions (superficial bites but no blood); 2 = moderate lesions (evidence of bites/teeth marks with fresh blood and/or infection and 3 = partial or total loss of the ear. The sum of scores on both left and right ears yielded a single score per pig per observation.

#### Flank lesions

Flank lesions (FL) were scored using a scale from 0 to 3 where 0 = no lesion; 1 = evidence of redness and/or a minor (superficial) lesion and/or blood (< 1cm); 2 = evidence of a larger lesion, blood and/or infection (> 1 cm) and 3 = evidence of an extensive (> 2. cm) or deep lesion, blood and/or infection. The sum of scores on both left and right flanks yielded a single score per pig per observation.

#### Limb lesions

Limb lesions (i.e. environmentally induced injuries) were scored according to their severity using the scoring method of de Koning [26] as modified by Calderón Díaz *et al*. [27]. Limb lesions were scored from 0 to 6 where 0 = normal; score 1 = redness, hairless (alopecia) or callus; score 2 = old scab; score 3 = wounds, swelling or bursitis; score 4 = severe wounds or severe swelling; score 6 = severe wounds plus severe swelling with inflammation. The sum of scores yielded a total limb lesion score for each pig.

#### Health deviations

All occurrences of health deviations in the focal pigs were also recorded as defined in the Welfare Quality® protocol [23]. Pigs were checked to assess thermal comfort (shivering and panting), diseases [pumping (heavy/laboured breathing), coughing, sneezing, twisted snouts, rectal prolapse, rupture/hernia, skin disorders, diarrhoea, nasal discharge] and lameness and were marked ‘1’ as present or ‘0’ as absent. Pigs were also checked for hematomas and claw damage. In addition, skin temperature was measured once a week using an infrared thermometer (Raytek Corporation GmbH, Berlin, Germany).

### Environmental measurements

Room temperature, CO_2_ concentrations and number of pigs per pen were recorded once per week during the group inspection. A portable sensor (Duomo Ltd., Worcestershire, UK) was used to measure room temperature and CO_2_ concentrations.

### Statistical analysis

Data were analysed using SAS v9.3 (SAS Inst. Inc., Cary, NC). Data were tested for normality before analysis using the Shapiro-Wilk test, checking skewness and kurtosis and examination of the normal plot. Health deviations were not included in the analysis at either focal or group level as there were few observations. For all tests, the criterion for statistical significance was established at *P* < 0.05 and statistical trends were reported 0.05 > *P* < 0.10.

#### Group measurements

Pen was considered the experimental unit. First and second weaner stages were analysed separately to account for the change of group composition between stages. Health deviations and flank lesions were not included in the analysis at group level as there were too few observations.

The proportion of animals with ear and tail lesions was analysed using binomial logistic regression in PROC GENMOD. ADG, ADFI and FCR were analysed using a general linear model in PROC GML; treatment was included as a fixed effect while initial body weight of each stage was included as a linear covariate. Mortalities and injections were analysed using Chi-square test in PROC FREQ. Head knock in the first weaner stage were analysed using linear mixed models in PROC MIXED. Head knocks in second stage, fighting, ear, tail and flank biting behaviour were analysed using generalized linear mixed model equation methods in PROC GLIMMIX. Treatment, inspection time and their interaction were included as fixed effects. Stocking density, body weight, room temperature and CO_2_ were included in the model as linear covariates. Group within treatment was included as a random effect. A Tukey-Kramer adjustment was used to account for multiple comparisons.

#### Focal pig measurements

Each individual pig was considered the experimental unit. Ear and flank lesions and health deviations were not included in the analysis as there were few observations. Additionally, of all the limb lesions recorded, calluses and swellings were the only two lesions included in the analysis as there were few observations for the other limb lesions.

Skin lesions and total limb lesion scores were analysed using generalized linear mixed model equation methods in PROC GLIMMIX. Tail lesions, calluses and swellings were re-classified as a binomial variable where 1 = presence and 0 = absence and were analysed using binomial logistic regression in PROC GENMOD. For all the variables studied, models included treatment, sex, inspection time and their interactions as fixed effects. Lesion scores at the start of the trial, stocking density, body weight and skin temperature were included as linear covariates. Pig within inspection time was included as a random variable. For skin lesions, Tukey-Kramer adjustment was used to account for multiple comparisons and results are presented as least square means ± SE. Results for logistic regression analysis are reported as odds ratios (OR) with the associated 95% confidence intervals (CI).

## Results

### Production data and parenteral administration of antibiotics

AB pigs had higher ADG (*P* = 0.02) and ADFI (*P* = 0.05) than NO pigs during the first weaner stage (Table 1); however, treatment had no effect on ADG and ADFI during the second weaner stage (*P* > 0.05; Table 1). There was no difference in FCR between AB and NO pigs in either the first or second weaner stage (*P* > 0.05, Table 1). Treatment had no effect on final body weight at the end of the second weaner stage (41.4±1.36 kg vs. 43.3±1.40 kg, NO and AB pigs respectively; *P* = 0.218) or on the mortality rate during the entire weaner stage (2.14% vs. 1.90%, NO and AB pigs respectively; *P* = 0.806). There was almost double the number of parenteral administrations of antibiotic (in individual doses) recorded in NO (105 doses) compared with AB (58 doses) pigs during the entire weaner stage for a total of 25% vs. 13.8% (NO and AB pigs, respectively) of the animals treated per each treatment (*P* < 0.001).

**Table 1:** Average daily gain, average daily feed intake and feed conversion ratio (mean ± standard error of the mean) for pigs provided with in-feed antibiotics (n = 420) and for pigs with no in-feed antibiotics (n = 420) during the first and the second weaner stages.

Variables	Treatment		*P*-value
	In-feed antibiotics	No in-feed antibiotics	
*First weaner stage*			
Average daily gain, g	402.2	435.6	0.018
	±18.20	±13.03	
Average daily feed intake, g	584.6	646.5	0.048
	±39.88	±28.83	
Feed conversion ratio	1.48	1.52	0.483
	±0.034	±0.032	
*Second weaner stage*			
Average daily gain, g	711.0	743.7	0.774
	±32.31	±42.58	
Average daily feed intake, g	1380.9	1440.2	0.589
	±29.29	±60.09	
Feed conversion ratio	1.95	1.95	0.944
	±0.054	±0.045	

### Skin lesions

No difference were found between AB and NO pigs for TBL (13.4 ± 0.39 vs. 12.5 ± 0.37), FBL (7.7 ± 0.23 vs. 7.3 ± 0.22), or RBL (5.5 ± 0.22 vs. 5.8 ± 0.26) scores of focal pigs (*P* > 0.05). Additionally, sex had no significant effect on the aforementioned scores (*P* > 0.05). All the other variables (skin temperature, stocking density, time, body weight and lesion score at the start of the trial) were significantly associated with TBL, FBL and RBL (S1 Table).

### Tail lesions

AB focal pigs tended to have a greater risk of TL compared with NO pigs (OR = 1.2; 95% CI = 0.96–1.63; *P =* 0.10). The same finding was recorded at group level where AB pigs tended to be more likely to have TL during the second weaner stage than NO pigs (OR = 1.7; 95% CI = 0.97–2.99; *P =* 0.05); however, treatment had no effect on TL during the first stage (*P* > 0.05). All the other variables (body and room temperature, CO_2_, stocking density, time, body weight and lesion score at the start of the trial) were associated with TL at both focal and group level (S2 and S3 Tables).

### Ear lesions

At group level treatment had no effect on EL during the first stage (*P* > 0.05); however AB pigs were less likely to have ear lesions during the second stage than NO pigs (OR = 0.46; 95% CI = 0.25–0.84; *P* < 0.05). Ear lesions increased as time progressed during the first weaner stage (*P* < 0.05) but there was no effect of time during the second weaner stage (*P* > 0.05). Stocking density, room temperature and body weight were significantly associated with EL, although associations were different between the two stages (S3 Table).

### Limb lesions

Treatment was not a significant source of variation for either calluses or swellings (*P* > 0.05) in focal pigs. However, males tended to be less likely to have swellings on the limbs than females (OR = 0.7; 95% CI = 0.58–1.05; *P* = 0.10). Although treatment had no effect on total limb lesion scores (*P* > 0.05), there was a sex effect (5.9 ± 0.12 vs. 5.5 ± 0.13, males and females respectively; *P* < 0.05).

### Environmental measurements

Room temperature ranged between 23.5 and 29.5 C° in the first weaner stage while it was between 16.2 and 23.1 C° in the second weaner stage. CO_2_ concentrations ranged between 880 and 3600 ppm in the first weaner stage and between 1100 and 3000 ppm in the second weaner stage; with an average of 1867 ppm over the entire weaner stage.

### Behaviour

#### Behaviours observed during the first weaner stage

The number of fights tended to be higher in AB (6.9 ± 0.91) than in NO (5.6 ± 0.72) groups (*P =* 0.09). The number of fights increased from the first to the second week and decreased in the following weeks (*P* < 0.05; S4 Table). Treatment and time had no effect on head knock behaviour (*P* > 0.05). Treatment had no effect on tail and flank biting behaviours (*P* > 0.05); however, ear biting was higher in AB (21.4 ± 2.15) than in NO (17.4 ± 1.61) groups (*P* < 0.05). The frequency of tail biting increased as time progressed (*P* > 0.01; S5 Table). Time was not a significant source of variation for ear and flank biting behaviours (*P* > 0.05). In pens with a higher frequency of flank biting the stocking density (*P* < 0.001), room temperature (*P* < 0.05) and CO_2_ concentrations (*P* < 0.01) were also higher than in pens where less flank biting was observed (S5 Table). Additionally, pigs in pens where a higher frequency of ear biting was observed were heavier (*P* < 0.01) and room temperature was higher (*P* < 0.001) than in pens where less ear biting was recorded (S4 and S5 Tables).

#### Behaviours observed during the second weaner stage

Treatment had no effect on number of fights (*P* > 0.05) while the number of head knocks tended to be higher in AB (5.1 ± 0.34) than in NO (4.3 ± 0.31) groups (*P =* 0.08). The number of fights and head knocks increased with time (*P* < 0.05; S4 Table). Treatment had no effect on tail, ear and flank biting behaviours (*P* > 0.05, S5 Table). However, tail (*P =* 0.07), ear (*P* < 0.05) and flank (*P =* 0.09) biting tended to increase from the 1^st^ to the 3^rd^ week and reduced in the last week (S5 Table). In pens with a higher frequency of ear biting, body weight (*P* < 0.05) and room temperature (*P* < 0.01) were lower than in pens with less ear biting (S5 Table). Additionally, pigs in pens where a higher frequency of flank biting was observed were heavier (*P* < 0.05; S5 Table) than in pens where less flank biting was seen and they tended to perform more tail biting behaviours when room temperature was higher (*P* = 0.06; S5 Table). Stocking density and CO_2_ concentrations were not significant sources of variation for these harmful behaviours (*P* > 0.05) (S4 and S5 Tables).

## Discussion

Measures of performance collected in this study showed that AB pigs grew at a faster rate and that their feed intake was higher than pigs without antibiotics in their diet reflecting the widely acknowledged growth promotion effects of antibiotics when fed at therapeutic levels as well as at lower levels [28]. In fact, these differences led to a 2 kg body weight difference (albeit non-significant) at the end of the second weaner stage. However, the novelty of this study lies in its exploration of the effect of removing prophylactic AB from pig feed on indicators of pig health and welfare as, to our knowledge, there is limited research in this area.

There was no difference between treatments in aggression related skin lesion scores of focal pigs. Hence, the tendency for AB pigs to perform more fighting behaviours in the first weaner stage and to perform more head knocks in the second weaner stage could be considered somewhat surprising [29]. However, Stukenborg [30] stated that body lesion scores are not always well correlated with aggressive behaviour which may help to explain our finding. Furthermore, the lesion scores were conducted at focal level and the finding regarding behaviour was based on observations conducted at group level. The performance differences described above only related to the first weaner stage which is when the differences in aggressive behaviour were also detected. Hence, the tendency for AB pigs to fight more may have been related to their greater motivation to eat and therefore increased competition for access to feed. Indeed, while feed was available *ad libitum*, not all pigs could feed simultaneously, thereby contributing to competition for access to feed. Unfortunately, during the behaviour observations no distinction was made between aggressive interactions for access to feed and for other reasons so it is not possible to confirm if this was the reason for the higher levels of aggression amongst pigs on the antibiotic treatment.

Also possible is that the differences in aggressive behaviour between pigs fed AB and those without were attributable to a disturbance of their gut microbiota. This is supported by recent studies which showed not only the deleterious effect of antibiotic treatments on the biodiversity and survival of microbiota in different species such as mice [31] and pigs [32], but also that such changes in microbiota can have a significant impact on brain development and behaviour including anxiety [31].

There was a reduced likelihood of having tail lesions in NO compared with AB pigs, both at focal and group level. This finding is somewhat in accordance with those of Alban *et al*. [33]. Although these authors found a reduction in tail bite infections in pigs after a reduction in AB use, they were unable to provide an explanation. The likely higher motivation to eat in AB pigs may have caused more attacks on the tail from behind as pigs competed for access to the feeder [34] and thereby contributed to the increased levels of tail damage recorded in AB pigs. However, there was no effect of treatment on the performance of tail biting behaviour. Differentiating between tail bites associated with competition for access to the feeder and those not associated with feeding could have helped to better explain the finding regarding the lower likelihood of tail lesions in NO pigs. Nevertheless, due to the nature and underlying motivations of harmful behaviours, it is sometimes difficult to establish the cause; Taylor *et al*. [35] explained how prolonged tail-in-mouth behaviour (i.e. a gentle manipulation of the tail) may not lead to an evident injury to the tail but could predispose those tails to damage for a future outbreak. Conversely, severe tail damage can be caused by short, sharp bites if an acute outbreak of tail biting erupts.

Surprisingly, the reduced likelihood of having ear lesions in AB pigs compared to NO pigs during the second weaner stage is in disagreement with the higher number of ear bites performed by AB pigs compared with NO pigs during the first weaner stage. In this study more ear biting behaviour was observed in pens with heavier pigs irrespective of treatment. This may explain the higher ear biting detected in AB pigs given that they had higher growth performance at least in the first weaner stage. Opportunistic infection of ear lesions by organisms such as streptococci and staphylococci may contribute to the multifactorial problem of ear necrosis [36]. Hence, it is plausible that medicated feed could have had a curative effect on ear lesions in the AB pigs which only become evident during the second weaner stage. A possible curative effect is supported by the fact that 13% of NO focal pigs had high ear lesion scores (score 3 for at least one ear) while only 5% AB of pigs were affected by the highest severity score.

There was almost double the amount of antibiotic injections administered to NO compared to AB pigs. This reflects the substitution of prophylactic antibiotic treatment in feed with targeted therapeutic treatment of sick individuals by farm personnel. Furthermore it indicates that NO pigs were more likely to show clinical signs of illness. However, there were no observed differences between the two treatments in health deviations recorded weekly as per the modified Welfare Quality^®^ protocol. Indeed few health deviations were detected in pigs in either treatment, and differences between treatments in limb lesions were not detected. Farm personnel were clearly vigilant in their care of the NO pigs such that they identified and treated pigs with early signs of clinical illness on a daily basis. This meant that no major disease problems arose for the NO pigs, there was no increase in their mortality rate and ultimately no health deviations were detected at the weekly health and welfare inspections. This is in accordance with the observations of Laine *et al*. [37]. Other authors also reported a lack of detrimental health effects following removal of in-feed antibiotics. In fact, Alban *et al*. [33] reported a significant reduction in chronic pericarditis and chronic pneumonia following a reduction in AB usage in line with governmental restrictions. The improvements in management and/or husbandry practices implemented on the farm at the start of the study also likely played a role in preventing the occurrence of health deviations. Hence, if good management systems and husbandry are in place, in-feed AB use could be reduced or even eliminated [16, 37, 38] and replaced by the targeted therapeutic treatment of sick individual pigs.

Provision of in-feed medication at weaning constitutes one of the main uses of antibiotics in pigs in the EU [7, 8, 11]. Moreover, extended periods of prophylactic use are common on farms with a high level of disease, such as the one selected for this study. This farm historically had high levels of disease and antibiotics were provided in the feed during the entire weaner stage (nine weeks), for 5 of every 7 days each week, as prescribed by the farm veterinary surgeon. This over/misuse of AB may have implications for the development of AB resistance due not only to the length of the treatment applied but also due to the potential lack of efficacy of the antibiotic treatment. Both the in-feed (TMS) and the parenteral (amoxicillin) medications are broad-spectrum antibiotics. Hence, they were not specific to the actual diseases present on the farm. In addition, under-dosage is likely in animals in the first week immediately post weaning due to low feed intake levels during this period. Unfortunately, extended periods of similar non-specific prophylactic use are common on many farms, especially if treatment patterns are not reviewed, and are not in accordance with the principles of prudent prescribing.

Withdrawal of in-feed medication as carried out for the NO pigs in this study would result in a c. 97% reduction in overall AB use measured as doses (i.e. 9 in-feed doses/pig during the entire weaner stage for a total of 3,780 doses withdrawn from NO pigs). Withdrawals of this magnitude would be of great benefit in reducing AB use in the pig sector. The results of this study showed that although in-feed medication may have a growth promotion effect with some consequences for pig behaviour and welfare, there were minimal production or health differences between them and the NO pigs. Furthermore, the lack of difference in FCR and mortality rate between AB and NO pigs in both weaner stages shows that the NO pigs were as efficient as the AB pigs. Nevertheless, the 2 kg difference in body weight between AB and NO pigs could be an important deterrent to farmers in reducing prophylactic in-feed AB usage.

**Fig 1:**
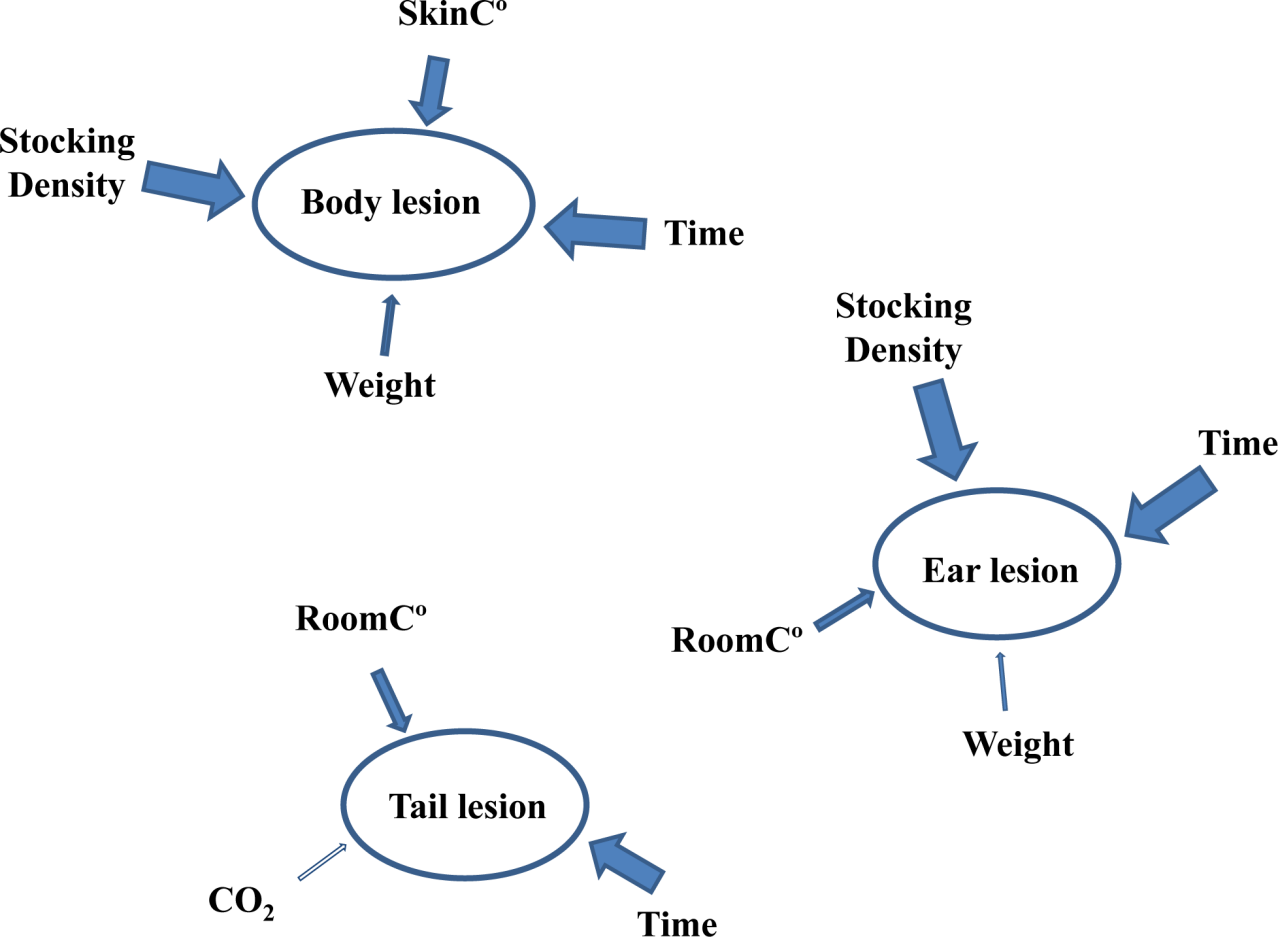
Representation of animal and environmental variables influencing body, tail and ear lesion scores in pigs during the first and second weaner stage. Animal Variables: Stocking density, SkinC° = skin temperature, Weight at both focal and group level. Environmental Variables: CO_2_, RoomC° = room temperature, Time = number of weeks (9 weeks) pigs were observed during the entire weaner stage. The larger the arrow, the larger the size of the effect of the variable on body, tail and ear lesion scores. Full arrows indicate significant effect (*P* < 0.05) while the empty arrow indicates a tendency (0.05 ≤ *P* ≤ 0.10).

Indeed, there is evidence of reluctance by some farmers to remove prophylactic in-feed AB usage from the diet of weaner pigs because of their belief that this practice is essential to preserve good pig welfare [39]. However, the findings of this study (see Fig 1) and other published research suggest that such a belief is unfounded as variables such as temperature [34, 40–43], stocking density [44–47], time and body weight [46, 48] had a greater influence on indicators of pig welfare. These findings were expected and they emphasise how many of the health and welfare problems in pigs are related to the physical and social environment [34, 49]. This is largely under the farmers control and means that a major effort should be applied to ensure adequate environmental conditions, since appropriate management systems and good housing are likely to be more beneficial in safeguarding pig welfare than the administration of prophylactic in-feed AB *per se*.

## Conclusion

Removing prophylactic antibiotics from the feed of weaner pigs had minimal effects on lesions related to welfare, health indicators and negative behaviours on this farm, suggesting that other farms using in-feed prophylactic antibiotics may be able to remove them without affecting pig health and welfare.

Differences in behaviour and welfare between pigs receiving in-feed antibiotics and those on un-medicated feed may have been mediated by higher growth rates in the former pigs. Variables such as time, environmental factors, skin temperature and bodyweight had more important effects on measures of behaviour and welfare than the presence or absence of prophylactic in-feed antibiotics. In-feed antibiotics seemed to play a role in mitigating the severity of ear lesions and their removal from the feed resulted in an increased need for therapeutic antibiotic treatment of individual animals showing clinical signs of disease. However, in spite of the fact that such pigs required twice the amount of parenteral treatments, the overall reduction in antibiotic use as measured in doses was more than 95%. Further research is necessary to evaluate the effect of withdrawal of in-feed prophylactic antibiotics on behaviour and welfare in other pig farms and to develop appropriate strategies to deal with the limited health and welfare problems (e.g. severity of ear lesions) that may arise in some animals.

## Supporting information

S1 Table Effect of time (weeks) and other variables (stocking density, lesion score at the start of the trial, body weight and skin temperature) on skin and limb lesion scores of focal pigs (n = 120 pigs). (DOCX)

S2 Table Effect of time (weeks) and other variables (stocking density, lesion score at the start of the trial, body weight and skin temperature) on the risk of tail lesions, calluses and swellings in focal pigs (n = 120). (DOCX)

S3 Table Effect of variables [stocking density, number of pigs affected at the start of each stage (week 1 of each stage) group weight, room temperature and CO_2_] on ear and tail lesions in groups of pigs during the first (n = 12 pens) and second (n = 24 pens) weaner stage. (DOCX)

S4 Table Effect of time (weeks) and other variables (stocking density, group weight, room temperature and CO_2_) on fighting and head knock behaviours in groups during the first (n = 12 pens) and second (n = 24 pens) weaner stage. (DOCX)

S5 Table Effect of time (weeks) and other variables (stocking density, group weight, room temperature and CO_2_) on tail, ear and flank biting behaviours in groups during the first (n = 12 pens) and second (n = 24 pens) weaner stage. (DOCX)
