# Correction: Do weaner pigs need in-feed antibiotics to ensure good health and welfare?

**DOI:** 10.1371/journal.pone.0189434

**Published:** 2017-12-07

**Authors:** Alessia Diana, Edgar G. Manzanilla, Julia A. Calderón Díaz, Finola C. Leonard, Laura A. Boyle

In Table 1, the headings In-feed antibiotics and No in-feed antibiotics are swapped in the second and third columns. The second column should be No in-feed antibiotics and the third should be In-feed antibiotics. Please see the corrected Table 1 here.

**Table 1:** Average daily gain, average daily feed intake and feed conversion ratio (mean ± standard error of the mean) for pigs provided with in-feed antibiotics (n = 420) and for pigs with no in-feed antibiotics (n = 420) during the first and the second weaner stages.

Variables	Treatment		*P*-value
	No In-feed antibiotics	In-feed antibiotics	
*First weaner stage*			
Average daily gain, g	402.2	435.6	0.018
	±18.20	±13.03	
Average daily feed intake, g	584.6	646.5	0.048
	±39.88	±28.83	
Feed conversion ratio	1.48	1.52	0.483
	±0.034	±0.032	
*Second weaner stage*			
Average daily gain, g	711.0	743.7	0.774
	±32.31	±42.58	
Average daily feed intake, g	1380.9	1440.2	0.589
	±29.29	±60.09	
Feed conversion ratio	1.95	1.95	0.944
	±0.054	±0.045	
