# Supplementary material for: Do weaner pigs need in-feed antibiotics to ensure good health and welfare?
Source: PLoS One. 2017 Oct 5;12(10):e0185622. doi: 10.1371/journal.pone.0185622 (PMC5628837; doi:10.1371/journal.pone.0185622)
Supplement: S1 Table — (DOCX) [file pone.0185622.s001.docx]

**S1 Table.**

|  | **Total body lesion score** | |  | **Front body lesion score** | | |  | **Rear body lesion score** | |  | | **Total limb**  **lesion score** | | |  |  |
| --- | --- | --- | --- | --- | --- | --- | --- | --- | --- | --- | --- | --- | --- | --- | --- | --- |
|  | **LS mean** | **SE** |  | **LS mean** | **SE** |  | | **LS mean** | **SE** | |  | | **LS mean** | **SE** | | |
| ***Week* ^**^** |  |  |  |  |  |  | |  |  |  | |  | | | |  |
| 1 | 9.0^e^ | 1.19 |  | 7.5^a,e^ | 1.02 |  | | 3.5^h^ | 0.51 |  | | 4.4^e^ 0.43 | | | |  |
| 2 | 3.1^d^ | 0.42 |  | 3.1^d^ | 0.44 |  | | 2.1^g^ | 0.38 |  | | 4.9^a,d,e^ 0.48 | | | |  |
| 3 | 5.9^c^ | 0.81 |  | 4.9^b,c^ | 0.70 |  | | 3.0^g,h^ | 0.45 |  | | 5.6^a,d^ 0.56 | | | |  |
| 4 | 10.4^e^ | 1.41 |  | 6.5^a,e^ | 0.92 |  | | 5.9^b,d,f^ | 0.86 |  | | 7.1^b,c^ 0.70 | | | |  |
| 5 | 14.0^a,b^ | 1.90 |  | 7.9^a,e^ | 1.10 |  | | 8.2^a,c,e^ | 1.19 |  | | 8.1^b^ 0.80 | | | |  |
| 6 | 17.8^b,e^ | 2.66 |  | 8.6^b,e^ | 1.30 |  | | 5.7^c,d,h^ | 0.66 |  | | 4.7^d,e^ 0.82 | | | |  |
| 7 | 26.3^a^ | 3.95 |  | 11.0^a,b,e^ | 1.68 |  | | 8.6^e,f^ | 0.98 |  | | 5.3^a,d,e^ 0.91 | | | |  |
| 8 | 29.3^a^ | 4.40 |  | 10.9^a,b,e^ | 1.67 |  | | 10.4^b,e,f^ | 1.18 |  | | 5.6^c,d,e^ 0.96 | | | |  |
| 9 | 31.3^a^ | 4.64 |  | 11.9^a^ | 1.78 |  | | 10.8^a,b^ | 1.20 |  | | 6.4^a,b,c^ 1.08 | | | |  |
| ***Stocking density (pigs × m^2^)^1^*** | 0.42 ± 0.103^*^ | |  | 0.31 ± 0.105^*^ | |  | | 0.18 ± 0.094**^^^** | |  | | NI | | | |  |
| ***Lesion score at the start of the trial^1^*** | 0.01 ± 0.002^**^ | |  | 0.01 ± 0.004**^^^** | |  | | 0.04 ± 0.008^**^ | |  | | 0.06 ± 0.006^**^ | | | |  |
| ***Body weight (kg)^1^*** | 0.03 ± 0.006^*^ | |  | 0.03 ± 0.006^*^ | |  | | 0.03 ± 0.004^*^ | |  | | 0.03 ± 0.004^**^ | | | |  |
| ***Skin temperature (Cº)^1^*** | 0.08 ± 0.019^*^ | |  | 0.05 ± 0.020^*^ | |  | | 0.08 ± 0.017^*^ | |  | | NI | | | |  |
| ^a,b,c,d^ Significant differences between predictor variables; *P* < 0.05; NI = not included in the model  ^1^ Results for continuous covariates presented as the regression coefficient ± SE;  ^*^*P* < 0.05; ^**^*P* < 0.001; **^^^** 0.05 ≤ *P* ≤ 0.10; | | | | | | | | | |  | |  | | | |  |
