# Supplementary material for: Do weaner pigs need in-feed antibiotics to ensure good health and welfare?
Source: PLoS One. 2017 Oct 5;12(10):e0185622. doi: 10.1371/journal.pone.0185622 (PMC5628837; doi:10.1371/journal.pone.0185622)
Supplement: S2 Table — (DOCX) [file pone.0185622.s002.docx]

**S2 Table.**

|  | |  | | **Tail lesion** | | | | | |  | | **Callus** | | | | | |  | | **Swelling** | | | | | |
| --- | --- | --- | --- | --- | --- | --- | --- | --- | --- | --- | --- | --- | --- | --- | --- | --- | --- | --- | --- | --- | --- | --- | --- | --- | --- |
|  | |  | |  | | **95% CI^***^** | | | |  | |  | | **95% CI^***^** | | | |  | |  | | **95% CI^***^** | | | |
|  | |  | | **OR^^^** | | **Lower** | | **Upper** | |  | | **OR^^^** | | **Lower** | | **Upper** | |  | | **OR^^^** | | **Lower** | | **Upper** | |
| ***Week* ^**^** |  | |  | |  | |  | |  | |  | |  | |  | |  | |  | |  | |  | |  |
| Reference = week 1 |  | |  | |  | |  | |  | |  | |  | |  | |  | |  | |  | |  | |  |
| 2 |  | | 0.6 | | 0.34 | | 1.23 | |  | | 1.1 | | 0.60 | | 2.03 | |  | | 2.8^a^ | | 1.33 | | 6.08 | |  |
| 3 |  | | 0.3^a^ | | 0.17 | | 0.59 | |  | | 1.2 | | 0.66 | | 2.30 | |  | | 6.5^a^ | | 3.20 | | 13.39 | |  |
| 4 | |  | | 0.3^a^ | | 0.16 | | 0.54 | |  | | 3.2^a^ | | 1.52 | | 6.76 | |  | | 10.0^a^ | | 4.92 | | 20.27 | |
| 5 | |  | | 0.2^a^ | | 0.14 | | 0.47 | |  | | 3.9^a^ | | 1.78 | | 8.73 | |  | | 12.4^a^ | | 6.11 | | 25.05 | |
| 6 | |  | | 0.1^a^ | | 0.05 | | 0.16 | |  | | 2.2^a^ | | 1.07 | | 4.40 | |  | | 3.0 | | 1.22 | | 7.55 | |
| 7 | |  | | 0.1^a^ | | 0.05 | | 0.19 | |  | | 4.3^a^ | | 1.89 | | 10.00 | |  | | 6.3^a^ | | 2.54 | | 15.76 | |
| 8 | |  | | 0.1^a^ | | 0.06 | | 0.20 | |  | | 9.2^a^ | | 3.05 | | 27.67 | |  | | 9.5^a^ | | 3.86 | | 23.58 | |
| 9 | |  | | 0.1^a^ | | 0.03 | | 0.10 | |  | | 7.2^a^ | | 2.60 | | 19.95 | |  | | 9.6^a^ | | 3.86 | | 23.69 | |
|  | |  | |  | |  | |  | |  | |  | |  | |  | |  | |  | |  | |  | |
| ***Stocking density (pigs × m^2^)^1^*** | |  | | NI | | | |  | |  | | NI | | | |  | |  | | NI | | | |  | |
|  | |  | |  | |  | |  | |  | |  | |  | |  | |  | |  | |  | |  | |
| ***Lesion score at the start of the trial^1^*** | |  | | 0.24 ± 0.090^*^ | | | |  | |  | | 1.31 ± 0.213^**^ | | | |  | |  | | 2.61 ± 0.423^**^ | | | |  | |
|  | |  | |  | |  | |  | |  | |  | |  | |  | |  | |  | |  | |  | |
| ***Body weight (kg)^1^*** | |  | | NI | | | |  | |  | | NI | | | |  | |  | | 0.12 ± 0.021^**^ | | | |  | |
|  | |  | |  | |  | |  | |  | |  | |  | |  | |  | |  | |  | |  | |
| ***Skin temperature (Cº)^1^*** | |  | | NI | | | |  | |  | | NI | | | |  | |  | | 0.11 ± 0.068 | | | |  | |
| ^1^ Results for continuous covariates presented as the regression coefficient ± SE; NI = not included in the model | | | | | | | | | | | | | | | | | | | | | | | | | |
| ^a^ Statistically different from the reference category, *P* < 0.05; ^*^*P* < 0.05; ^(a)^ Tend to be different from the reference category, (0.05 ≤ *P* ≤ 0.10);  **^^^** OR = odds ratios; ^***^CI = 95% confidence interval; ^**^*P* < 0.001; | | | | | | | | | | | | | | | | | | | | | | | | | |
