# Supplementary material for: Do weaner pigs need in-feed antibiotics to ensure good health and welfare?
Source: PLoS One. 2017 Oct 5;12(10):e0185622. doi: 10.1371/journal.pone.0185622 (PMC5628837; doi:10.1371/journal.pone.0185622)
Supplement: S3 Table — (DOCX) [file pone.0185622.s003.docx]

**S3 Table.**

|  |  | | **1^st^ stage** | | | | |  | **2^nd^ stage** | | | | | |
| --- | --- | --- | --- | --- | --- | --- | --- | --- | --- | --- | --- | --- | --- | --- |
|  |  | | **Ear lesion** |  | **Tail lesion** | | |  | **Ear lesion** | | |  |  | **Tail lesion** |
| ***Stocking density (pigs × m^2^)^1^*** | |  | -1.65 ± 1.13**^#^** |  | NI | | |  | -2.36 ± 0.675^*^ | | |  |  | NI |
| ***No. pigs affected at the start of the stage^1^*** | |  | NI |  | 6.48 ± 1.82^**^ | | |  | 6.95 ± 1.044^*^ | | |  |  | NI |
| ***Group weight (kg)^1^*** | |  | NI |  | NI | | |  | 0.02 ± 0.005^**^ | | |  |  | NI |
| ***Room temperature (Cº)^1^*** | |  | 0.25 ± 0.08^*^ |  | -0.27 ± 0.08^**^ | | |  | -0.40 ± 0.140^*^ | | |  |  | NI |
| ***CO_2_^1^*** | |  | NI |  | NI | | |  |  | NI | | |  | -0.001 ± 0.0003**^#^** |
| ^1^ Results for continuous covariates presented as the regression coefficient ± SE; | | | | | |  |  | | | |  | |  |  |
| ^a^ Statistically different from the reference category, *P* < 0.05; ^(a)^ Tend to be different from the reference category, (0.05 ≤ *P* ≤ 0.10);  ^*^*P* < 0.05; ^**^*P* < 0.01; ^#^ 0.05 ≤ *P* ≤ 0.10; NI = not included in the model | | | | | | | | | | | | | | |
