# Supplementary material for: Do weaner pigs need in-feed antibiotics to ensure good health and welfare?
Source: PLoS One. 2017 Oct 5;12(10):e0185622. doi: 10.1371/journal.pone.0185622 (PMC5628837; doi:10.1371/journal.pone.0185622)
Supplement: S4 Table — (DOCX) [file pone.0185622.s004.docx]

**S4 Table.**

|  | **Fighting** | | | |  | | | **Head knock** | | | | | | |  |
| --- | --- | --- | --- | --- | --- | --- | --- | --- | --- | --- | --- | --- | --- | --- | --- |
|  | **1^st^ stage** | | **2^nd^ stage** | | |  | | **1^st^ stage** | | | | **2^nd^ stage** | | |  |
|  | **LS mean** | **SE** | **LS mean** | **SE** |  | | **LS mean** | | **SE** | | **LS mean** | | | **SE** |  |
| **Week** **^*^** |  |  |  |  |  | |  | |  | |  | | |  |  |
| 1 | 4.1^b^ | 0.99 | 1.1^b^ | 0.21 |  | | NI | | NI | | 2.7^c^ | | | 0.42 |  |
| 2 | 10.1^a^ | 1.98 | 2.1^a^ | 0.30 |  | | NI | | NI | | 6.9^a^ | | | 0.54 |  |
| 3 | 7.1^b,a^ | 1.61 | 1.1^b^ | 0.22 |  | | NI | | NI | | 4.3^b,c^ | | | 0.42 |  |
| 4 | 5.0^b,a^ | 1.21 | 2.0^b,a^ | 0.29 |  | | NI | | NI | | 5.9^b,a^ | | | 0.53 |  |
|  |  |  |  |  |  | |  | |  | |  | | |  |  |
| ***Stocking density (pigs × m^2^)^1^*** | NI | | NI | | |  | | 19.71 ± 10.642**^^^** | | | | NI | | |  |
|  |  |  |  |  |  | |  | |  | |  | | |  |  |
| ***Group weight (kg)^1^*** | 0.01 ± 0.002**^^^** | | NI | | |  | | -0.08 ± 0.044**^^^** | | | | -0.01 ± 0.001**^^^** | | |  |
|  |  |  |  |  |  | |  | |  | |  | | |  |  |
| ***Room temperature (Cº)^1^*** | 0.13 ± 0.060^*^ | | NI | | |  | | NI | | | | -0.12 ± 0.033^**^ | | |  |
|  |  |  |  |  |  | |  | |  | |  | | |  |  |
| ***CO_2_ ^1^*** | 0.0003 ± 0.0001^*^ | | NI | | |  | | NI | | | | NI | | |  |
| ^a,b,c^ Significant differences between predictor variables, *P* < 0.05;  ^(a)^ tendency between predictor variables, (0.05 ≤ *P* ≤ 0.10); | | | | | | | | | | | | |  |  |  |
| ^1^ Results for continuous covariates presented as the regression coefficient ± SE; | | | | | | | | | | | | | | | |
| ^*^*P* < 0.05; ^**^*P* < 0.01; **^^^** 0.05 ≤ *P* ≤ 0.10; NI = not included in the model | | | | | | | | | |  |  |  |  |  |  |
