# Supplementary material for: Do weaner pigs need in-feed antibiotics to ensure good health and welfare?
Source: PLoS One. 2017 Oct 5;12(10):e0185622. doi: 10.1371/journal.pone.0185622 (PMC5628837; doi:10.1371/journal.pone.0185622)
Supplement: S5 Table — (DOCX) [file pone.0185622.s005.docx]

**S5 Table.**

|  | **Tail Biting** | | | |  | **Ear Biting** | | | | | | | | | | | |  | **Flank Biting** | | | | | |
| --- | --- | --- | --- | --- | --- | --- | --- | --- | --- | --- | --- | --- | --- | --- | --- | --- | --- | --- | --- | --- | --- | --- | --- | --- |
|  | **1^st^ stage** |  | **2^nd^ stage** | |  | **1^st^ stage** |  | | | **2^nd^ stage** | | | | | | | |  | **1^st^ stage** |  | **2^nd^ stage** | | | |
|  | **LS mean** | **SE** | **LS mean** | **SE** |  | **LS mean** | **SE** | | | **LS mean** | | | | | **SE** | | |  | **LS mean** | **SE** | **LS mean** | | **SE** |  |
| ***Week*** |  |  |  |  |  |  |  | | |  | | | | |  | | |  |  |  |  |  | | |
| 1 | 0.3^b^ | 0.17 | 0.1^b^ | 0.05 |  | NI | NI | | | 2.2^b,a^ | | | | | 0.50 | | |  | 0.8^a^ | 0.45 | 0.3^a^ | 0.10 | | |
| 2 | 2.2^a^ | 0.42 | 0.4^b,a^ | 0.13 |  | NI | NI | | | 2.8^a^ | | | | | 0.36 | | |  | 1.3^a^ | 0.67 | 0.6^a^ | 0.15 | | |
| 3 | 1.4^b,a^ | 0.34 | 0.5^a^ | 0.17 |  | NI | NI | | | 2.4^a^ | | | | | 0.32 | | |  | 3.4^a^ | 1.47 | 0.8^a^ | 0.18 | | |
| 4 | 2.3^a^ | 0.44 | 0.3^b,a^ | 0.12 |  | NI | NI | | | 1.5^b^ | | | | | 0.27 | | |  | 0.9^a^ | 0.50 | 0.7^a^ | 0.17 | | |
|  |  |  |  |  |  |  |  | | |  | | | | |  | | |  |  |  |  |  | | |
| ***Stocking density (pigs × m^2^)^1^*** | NI | | NI | |  | NI | | | | NI | | | | | | | |  | 4.91± 0.893^***^ | | NI | | | |
|  |  |  |  |  |  |  |  | | |  | | | | |  | | |  |  |  |  |  | | |
| ***Group weight (kg)^1^*** | NI | | NI | |  | 0.01 ± 0.001^**^ | | | | -0.01 ± 0.001^*^ | | | | | | | |  | NI | | 0.01 ± 0.003^*^ | | | |
|  |  |  |  |  |  |  |  | | |  | | | | |  | | |  |  |  |  |  | | |
| ***Room temperature (Cº)^1^*** | NI | | 0.26 ± 0.140**^^^** | |  | 0.23 ± 0.042^***^ | | | | -0.35 ± 0.088^**^ | | | | | | | |  | 0.34 ± 0.128^*^ | | NI | | | |
|  |  |  |  |  |  |  |  | | |  | | | | |  | | |  |  |  |  |  | | |
| ***CO_2_ ^1^*** | NI | | NI | |  | NI | | | | 0.001 ± 0.0002 | | | | | | | |  | 0.001 ± 0.0002^**^ | | NI | | | |
| ^a,b,c^ Significant differences between predictor variables; *P* < 0.05 | | | | | | | | | |  | |  | | | |  |  |  |  |  |  |  |  |  |
| ^1^ Results for continuous covariates presented as the regression coefficient ± SE | | | | | | | | | | | | | | | |  |  |  |  |  |  |  |  |  |
| ^*^*P* < 0.05; ^**^*P* < 0.01; ^***^*P* < 0.001; **^^^** 0.05 ≤ *P* ≤ 0.10; NI = not included in the model | | | | | | |  |  |  | |  | |  |  | | |  |  |  |  |  |  |  |  |
