# Supplementary material for: Correction: Correction: Do weaner pigs need in-feed antibiotics to ensure good health and welfare?
Source: PLoS One. 2018 Feb 22;13(2):e0193505. doi: 10.1371/journal.pone.0193505 (PMC5823442; doi:10.1371/journal.pone.0193505)
Supplement: S2 File — (PDF) [file pone.0193505.s002.pdf]

PLOS ONE

CORRECTION

Correction: Do weaner pigs need in-feed antibiotics to ensure good health and welfare?

Alessia Diana, Edgar G. Manzanilla, Julia A. Calderón Díaz, Finola C. Leonard, Laura A. Boyle

In Table 1, the headings In-feed antibiotics and No in-feed antibiotics are swapped in the second and third columns. The second column should be No in-feed antibiotics and the third should be In-feed antibiotics. Please see the corrected Table 1 here.

Check for updates

OPEN ACCESS

Citation: Diana A, Manzanilla EG, Calderón Díaz JA, Leonard FC, Boyle LA (2017) Correction: Do weaner pigs need in-feed antibiotics to ensure good health and welfare? PLoS ONE 12(12): e0189434. https://doi.org/10.1371/journal.pone.0189434

Published: December 7, 2017

Copyright: © 2017 Diana et al. This is an open access article distributed under the terms of the Creative Commons Attribution License, which permits unrestricted use, distribution, and reproduction in any medium, provided the original author and source are credited.

Table 1. Average daily gain, average daily feed intake and feed conversion ratio (mean ± standard error of the mean) for pigs provided with in-feed antibiotics (n = 420) and for pigs with no in-feed antibiotics (n = 420) during the first and the second weaner stages.

Variables	Treatment		*P*-value
	No In-feed antibiotics	In-feed antibiotics	
First weaner stage			
Average daily gain, g	402.2	435.6	0.018
	±18.20	±13.03	
Average daily feed intake, g	584.6	646.5	0.048
	±39.88	±28.83	
Feed conversion ratio	1.48	1.52	0.483
	±0.034	±0.032	
Second weaner stage			
Average daily gain, g	711.0	743.7	0.774
	±32.31	±42.58	
Average daily feed intake, g	1380.9	1440.2	0.589
	±29.29	±60.09	
Feed conversion ratio	1.95	1.95	0.944
	±0.054	±0.045	

https://doi.org/10.1371/journal.pone.0189434.t001

Reference

1. Diana A, Manzanilla EG, Calderón Díaz JA, Leonard FC, Boyle LA (2017) Do weaner pigs need in-feed antibiotics to ensure good health and welfare? PLoS ONE 12(10): e0185622. https://doi.org/10.1371/journal.pone.0185622 PMID: 28982114
